# Supplementary material for: European List of Essential Medicines for Medical Education: a protocol for a modified Delphi study
Source: BMJ Open. 2021 May 4;11(5):e045635. doi: 10.1136/bmjopen-2020-045635 (PMC8098946; doi:10.1136/bmjopen-2020-045635)
Supplement: Supplementary data [file bmjopen-2020-045635supp001.pdf]

Appendix 1

All guideline medicines to treat the 'essential diseases', ordered alphabetically. When note down more than once, means that there is more than one indication.

Acenocoumarol
Acenocoumarol
Acetylsalicylic acid
Acetylsalysilic acid
Alendronic acid
Aluminium acetotartrate -eardrip
Aluminum hydroxide/magnesium hydroxide
Amitriptyline
Amlodipine
Amoxicillin
Amoxicillin
Amoxicillin
Amoxicillin
Amoxicillin/clavulanate
Amoxicillin/clavulanate
Amoxicillin/clavulanate
Atorvastatin
Azithromycin
Azithromycin
Azithromycin
Azithromycin
Azithromycin
Beclometasone
Betamethasone
Butylscopolamine
Carbasalate calcium
Chloramphenicol
Ciprofloxacin
Citalopram
Clarithromycin
Clarithromycin
Claritromycin
Clemastine
Clemastine
Clomipramine
Clopidogrel
Clopidogrel
Clotrimazole
Codeine
Colchicine
Colecalciferol (with/without) calcium
Desloratadine
Desloratadine
Desloratadine
Desogestrel (oral)
Dexamethason
Dexamethason
Diazepam
Diclofenac
Diclofenac
Diclofenac
Diclofenac
Digoxin
Diltiazem
Diltiazem
Dipyridamole
Domperidone
Domperidone
Doxycycline
Doxycycline
Doxycycline
Doxycycline
Emollients (e.g. Cremor vaselini cetomacrogolis)
Emollients (e.g. Cremor vaselini cetomacrogolis)
Emollients (e.g. Cremor vaselini cetomacrogolis)
Enalapril
Epinephrine (adrenaline)
Epinephrine (adrenaline)
Esomeprazole
Ethinylestradiol/levonorgestrel (oral)
Etonogestrel (s.c. Implant)
Ferrous fumarate
Flucloxacillin
Flucloxacillin
Flucloxacillin
Fluconazole
Fluticasone
Fluticasone
Folic acid (vitamin b9)
Formoterol
Fosfomycin
Fusidic acid
Gliclazide
Glucagon
Glucose solution
Glyceryl trinitrate (nitroglycerine)
Haloperidol
Hydrochlorothiazide
Hydrocortisone
Hydrocortisone
Hydrocortisone/acetic acid

Hydroxocobalamin (vitamin b12)
Ibuprofen
Ibuprofen
Ibuprofen
Ibuprofen
Influenza vaccine
Insulin aspart
Insulin glargine
Ipratropium bromide
Ipratropium bromide
Isosorbide dinitrate
Isosorbide mononitrate
Ispaghula (psylla seeds)
Lactulose
Levocabastine
Levocetirizine
Levocetirizine
Levocetirizine
Levonorgestrel (iud)
Levothyroxine
Lidocaine creme
Lisinopril
Loperamide
Loperamide
Lorazepam
Losartan
Macrogol
Macrogol
Mebeverine
Medroxyprogesterone (s.c. Depot)
Menthol in aqueous cream
Menthol in aqueous cream
Metformin
Metocloperamide
Metocloperamide
Metoprolol
Metoprolol
Metronidazole
Miconazol
Midazolam (i.m.)
Mometasone
Nadroparin
Nadroparin
Naproxen
Naproxen
Naproxen
Naproxen
Nitrofurantoin
Omeprazole
Omeprazole, pantoprazole
Omeprazole, pantoprazole, esomeprazole
Ors (oral rehydration solution)
Ors (oral rehydration solution)
Oxazepam
Oxycodone
Pantoprazole
Paracetamol
Paracetamol
Paroxetine
Perindopril
Pheneticillin
Pheneticillin
Pheneticillin
Phenprocoumon
Phenprocoumon
Prednisolone
Prednisolone
Prednisolone
Prensisolone
Propranolol
Ranitidine
Risedronic acid
Rosuvastatin
Salbutamol
Salbutamol
Salbutamol
Salbutamol
Salbutamol
Salbutamol
Salmeterol
Simvastatin
Sodium chloride solution
Spironolactone
Temazepam
Tetanus toxoid
Thiamine (vit b1)
Tiotropium bromide
Tramadol
Triamcinolone
Triamcinolone/acetic acid
Trimethoprim
Trimethoprim/polymyxine b
Trimethoprim/sulfamethoxazole
Trimethoprim/sulfamethoxazole
Trimethoprim/sulfamethoxazole
Verapamil
Verapamil
Xylometazoline nasalspray
Xylometazoline nasalspray
Zolpidem
