# Supplementary material for: European List of Essential Medicines for Medical Education: a protocol for a modified Delphi study
Source: BMJ Open. 2021 May 4;11(5):e045635. doi: 10.1136/bmjopen-2020-045635 (PMC8098946; doi:10.1136/bmjopen-2020-045635)
Supplement: Supplementary data [file bmjopen-2020-045635supp002.pdf]

List of medicines	Route of administration	Route of administration	Route of administration	Route of administration
Miconazole	Oral			
Aluminium hydroxide/magnesium hydroxide	Oral			
Magnesium hydroxide	Oral			
Ranitidine	Oral			
Misoprostol	Oral			
Omeprazole	Oral			
Pantoprazole	Oral			
Esomeprazole	Oral	IV		
Mebeverine	Oral			
Atropine	IV			
Butylscopolamine	Oral			
Metoclopramide	Oral	IV	Rectal	
Domperidone	Oral			
Ondansetron	Oral	IV	Rectal	
Bisacodyl	Oral	Rectal		
Ispaghula husk	Oral			
Lactulose	Oral			
Macrogol	Oral			
Nystatin	Oral			
Oral rehydration solution (ORS)	Oral			
Loperamide	Oral			
Insulin	SC			
Insulin aspart	SC			
Protamine (novomix)	SC			
Insulin glargine	SC			
Metformin	Oral			
Gliclazide	Oral			
Glimepiride	Oral			
Acarbose	Oral			
Pioglitazone	Oral			
Sitagliptin	Oral			
Linagliptin	Oral			
Exenatide	SC			
Liraglutide	SC			
Canagliflozin	Oral			
Empagliflozin	Oral			
Repaglinide	Oral			
Cholecalciferol	Oral			
Thiamine (vit B1)	Oral	IV		
Calcium/vitamin D	Oral			
Potassium chloride	Oral			
Fenprocoumon	SC			
Acenocoumarol	SC			
Heparin	IV			

Enoxaparin	SC	
Nadroparin	SC	
Clopidogrel	Oral	
Dipyridamole	Oral	
Carbasalate calcium	Oral	
Prasugrel	Oral	
Dabigatran	Oral	
Rivaroxaban	Oral	
Apixaban	Oral	
Fondaparinux	SC	IV
Tranexamic acid	Oral	IV
Vitamin K	Oral	IV
Prothrombin complex concentrate	IV	
Ferrous fumarate	Oral	
Hydroxocobalamin (vitamin B12)	SC	IM
Folic acid (vitamin B9)	Oral	
Glucose solution	IV	
Potassium chloride	IV	
Digoxin	Oral	IV
Flecainide	Oral	
Amiodarone	Oral	IV
Epinephrine (adrenaline)	IM	
Nitroglycerine	SL	
Isosorbide dinitrate	Oral	SL
Isosorbide mononitrate	Oral	
Methyldopa	Oral	
Hydrochlorothiazide	Oral	
Chlorthalidone	Oral	
Furosemide	Oral	IV
Bumetanide	Oral	IV
Spironolactone	Oral	
Propranolol	Oral	
Sotalol	Oral	
Metoprolol	Oral	IV
Atenolol	Oral	
Bisoprolol	Oral	
Labetolol	Oral	IV
Amlodipine	Oral	
Nifedipine	Oral	
Barnidipine	Oral	
Lercanidipine	Oral	
Nicardipine	IV	
Verapamil	Oral	IV
Diltiazem	Oral	
Enalapril	Oral	
Lisinopril	Oral	

Perindopril	Oral	
Ramipril	Oral	
Fosinopril	Oral	
Losartan	Oral	
Valsartan	Oral	
Irbesartan	Oral	
Candesartan	Oral	
Simvastatin	Oral	
Pravastatin	Oral	
Atorvastatin	Oral	
Rosuvastatin	Oral	
Gemfibrozil	Oral	
Colestyramine	Oral	
Ezetimibe	Oral	
Evolocumab	SC	
Miconazole	Cutaneous	
Emollients (e.g. Cremor vaseline cetomacrogolis)	Cutaneous	
Zinc oxide	Cutaneous	
Petroleum jelly	Cutaneous	
Lidocaine creme	Cutaneous	
Menthol in hydrophilic ointment	Cutaneous	
Fusidic acid	Cutaneous	
Hydrocortisone	Cutaneous	
Triamcinolone	Cutaneous	
Betamethasone	Cutaneous	
Mometasone	Cutaneous	
Clobetasole	Cutaneous	
Clotrimazole	Cutaneous	
IUD with progestogen	IUD	
Ethinylestradiol/levonorgestrel	Oral	
Medroxyprogesterone (s.c. Depot)	SC	
Etonogestrel (s.c. Implant)	SC	
Desogestrel	Oral	
Levonorgestrel	Oral	
Estradiol	Oral	
Oxybutynin	Oral	
Solifenacin	Oral	
Tamsulosin	Oral	
Finasteride	Oral	
Betamethasone	Oral	IV
Dexamethasone	Oral	IV
Prednisolone	Oral	IV
Hydrocortisone	Oral	IV
Levothyroxine	Oral	
Glucagon	IM	

Doxycycline	Oral			
Amoxicillin	Oral	IV		
Feneticillin	Oral	IV		
Flucloxacillin	Oral	IV		
Amoxicillin/clavulanic acid	Oral	IV		
Meropenem	IV			
Trimethoprim	Oral			
Co-trimoxazole	Oral	IV		
Trimethoprim/sulfametoxazole	Oral	IV		
Erythromycin	Oral	IV		
Clarithromycin	Oral	IV		
Azithromycin	Oral	IV		
Gentamycin	IV			
Ciprofloxacin	Oral	IV		
Vancomycin	IV			
Fusidic acid	Oral			
Nitrofurantoin	Oral			
Fosfomycin	Oral			
Fluconazole	Oral	IV		
Rifampicin	Oral	IV		
Acyclovir	Oral	IV		
Valaciclovir	Oral			
Oseltamivir	Oral			
Tetanus toxoid	IM			
Methotrexate	Oral	IV	IM	SC
Diclofenac	Oral			
Ibuprofen	Oral			
Naproxen	Oral			
Ibuprofen	Cutaneous			
Diclofenac	Cutaneous			
Allopurinol	Oral			
Colchicine	Oral			
Alendronic acid	Oral			
Risedronic acid	Oral			
Denosumab	SC			
Fentanyl	IV			
Lidocaine	SC			
Epinephrine (adrenaline)	SC			
Morphine	Oral	IV	SC	
Oxycodone	Oral	SC		
Fentanyl	Oral	Cutaneous	Nasal	SL
Tramadol	Oral	Rectal		
Piritramide	SC			
Acetylsalicylic acid	Oral	IV		
Calcium carbasalate	Oral	IV		
Paracetamol	Oral	IV	Rectal	

Sumatriptan	Oral	Nasal	SC
Valproic acid	Oral	IV	
Levetiracetam	Oral	IV	
Pregabalin	Oral	IV	
Haloperidol	Oral	IV	
Clozapine	Oral		
Olanzapine	Oral		
Quetiapine	Oral		
Lithium	Oral		
Risperidone	Oral	IM	
Diazepam	Oral	Rectal	
Oxazepam	Oral		
Lorazepam	Oral	IM	IV
Temazepam	Oral		
Midazolam	Oral	IV	
Zolpidem	Oral		
Clomipramine	Oral		
Amitriptyline	Oral		
Nortriptyline	Oral		
Fluoxetine	Oral		
Citalopram	Oral		
Paroxetine	Oral		
Sertraline	Oral		
Escitalopram	Oral		
Mirtazapine	Oral		
Venlafaxine	Oral		
Metronidazole	Oral	IV	
Xylometazoline	Nasal		
Levocabastine	Ocular		
Beclomethasone	Nasal		
Budesonide	Nasal		
Mometasone	Nasal		
Fluticasone	Nasal		
Salbutamol	Inhalation		
Salmeterol	Inhalation		
Formoterol	Inhalation		
Beclometasone	Inhalation		
Budesonide	Inhalation		
Fluticasone	Inhalation		
Ciclesonide	Inhalation		
Ipratropium bromide	Inhalation		
Tiotropium bromide	Inhalation		
Tiotropium	Inhalation		
Codeine	Oral		
Clemastine	Oral	IV	
Meclozine	Oral		

Levocetirizine	Oral	
Desloratadine	Oral	
Chloramphenicol	Ocular	
Fusidic acid	Ocular	
Erythromycin	Ocular	
Trimethoprim/polymyxin B	Ocular	
Dexamethasone (Ocular)	Ocular	
Prednisolone (Ocular)	Ocular	
Levocabastine	Ocular	
Aluminium acetate	Ear	
Miconazole	Oral	
Hydrocortisone/acetic acid	Ear	
Triamcinolone/acetic acid	Ear	
Protamine	IV	
Naloxone	IV	
Flumazenil	IV	
Ticagrelor	Oral	IV
Cefuroxime	IV	
Cefotaxime	IV	
Ceftriaxone	IV	
Ceftazidime	IV	
Clindamycin	Oral	IV
Sodium chloride	IV	
Ringer’s lactate	IV	
