# Supplementary material for: European List of Essential Medicines for Medical Education: a protocol for a modified Delphi study
Source: BMJ Open. 2021 May 4;11(5):e045635. doi: 10.1136/bmjopen-2020-045635 (PMC8098946; doi:10.1136/bmjopen-2020-045635)
Supplement: Author's manuscript [file bmjopen-2020-045635.draft_revisions.pdf]

BMJ Open is committed to open peer review. As part of this commitment we make the peer review history of every article we publish publicly available.

When an article is published we post the peer reviewers' comments and the authors' responses online. We also post the versions of the paper that were used during peer review. These are the versions that the peer review comments apply to.

The versions of the paper that follow are the versions that were submitted during the peer review process. They are not the versions of record or the final published versions. They should not be cited or distributed as the published version of this manuscript.

BMJ Open is an open access journal and the full, final, typeset and author-corrected version of record of the manuscript is available on our site with no access controls, subscription charges or pay-per-view fees (http://bmjopen.bmj.com).

If you have any questions on BMJ Open's open peer review process please email info.bmjopen@bmj.com

BMJ Open

The European List of Essential Medicines for Medical Education, a Protocol for a Modified Delphi Study

Journal:	*BMJ Open*
Manuscript ID	bmjopen-2020-045635
Article Type:	Protocol
Date Submitted by the Author:	08-Oct-2020
Complete List of Authors:	Donker, Erik; Amsterdam UMC Locatie VUmc, Department of Internal Medicine, section Pharmacotherapy<br>Brinkman, David; Amsterdam UMC Locatie VUmc, Department of Internal Medicine, section Pharmacotherapy<br>Richir, Milan; Amsterdam UMC Locatie VUmc, Department of Internal Medicine, section Pharmacotherapy<br>Papaioannidou, Paraskevi; Aristotle University of Thessaloniki Faculty of Health Sciences, Department of Pharmacology, School of Medicine<br>Likic, Robert; University of Zagreb School of Medicine; University Hospital Centre Zagreb, Unit of Pharmacology<br>Sanz, Emilio ; Universidad de La Laguna, School of Health Science<br>Christiaens, Thierry; Ghent University, Department of Clinical Pharmacology<br>Costa, Joao; University of Lisbon, Department of Pharmacology and Clinical Pharmacology<br>De Ponti, Fabrizio; University of Bologna, Department of Medical and Surgical Sciences<br>Gatti, Milo; University of Bologna, Department of Medical and Surgical Sciences<br>Böttiger, Ylva; Linkopings universitet, Department of Medical and Health Sciences<br>Kramers, Cornelis; Radboud University Medical Center, Department of Pharmacology-Toxicology<br>Garners, Sarah; WHO Regional Office for Europe, Health Technologies and Pharmaceuticals Programme<br>Pandit, Rahul; University Medical Centre Utrecht Brain Centre, Department of Translational Neuroscience<br>van Agtmael, Michiel; Amsterdam UMC Locatie VUmc, Department of Internal Medicine, section Pharmacotherapy<br>Tichelaar, Jelle; Amsterdam UMC Locatie VUmc, Department of Internal Medicine, section Pharmacotherapy
Keywords:	EDUCATION & TRAINING (see Medical Education & Training), MEDICAL EDUCATION & TRAINING, THERAPEUTICS

I, the Submitting Author has the right to grant and does grant on behalf of all authors of the Work (as defined in the below author licence), an exclusive licence and/or a non-exclusive licence for contributions from authors who are: i) UK Crown employees; ii) where BMJ has agreed a CC-BY licence shall apply, and/or iii) in accordance with the terms applicable for US Federal Government officers or employees acting as part of their official duties; on a worldwide, perpetual, irrevocable, royalty-free basis to BMJ Publishing Group Ltd ("BMJ") its licensees and where the relevant Journal is co-owned by BMJ to the co-owners of the Journal, to publish the Work in this journal and any other BMJ products and to exploit all rights, as set out in our licence.

The Submitting Author accepts and understands that any supply made under these terms is made by BMJ to the Submitting Author unless you are acting as an employee on behalf of your employer or a postgraduate student of an affiliated institution which is paying any applicable article publishing charge ("APC") for Open Access articles. Where the Submitting Author wishes to make the Work available on an Open Access basis (and intends to pay the relevant APC), the terms of reuse of such Open Access shall be governed by a Creative Commons licence – details of these licences and which Creative Commons licence will apply to this Work are set out in our licence referred to above.

Other than as permitted in any relevant BMJ Author's Self Archiving Policies, I confirm this Work has not been accepted for publication elsewhere, is not being considered for publication elsewhere and does not duplicate material already published. I confirm all authors consent to publication of this Work and authorise the granting of this licence.

For peer review only

The European List of Essential Medicines for Medical Education, a Protocol for a Modified Delphi Study

Corresponding and principle author
Erik (Martin) Donker
Amsterdam UMC, location VUmc
Dept. Internal medicine, Section Pharmacotherapy
De Boelelaan 1117
1081HV Amsterdam
The Netherlands.
e.donker@amsterdamumc.nl

Co-authors and affiliations
David J. Brinkman[1,2]
Milan C. Richir[1,2]
Paraskevi Papaioannidou[3]
Robert Likic[4]
Emilio J Sanz[5]
Thierry Christiaens[6]
João N Costa[7]
Fabrizio De Ponti[8]
Milo Gatti[8]
Ylva Böttiger[9]
Cornelis Kramers[10]
Sarah Garner[11]
Rahul Padit[12]
Michiel A van Agtmael[1,2]
Jelle Tichelaar[1,2]

1 Department of Internal Medicine, section Pharmacotherapy, Amsterdam UMC, location VUmc, Amsterdam, Netherlands

2 Research and Expertise Centre in Pharmacotherapy Education (RECIPE), Amsterdam, The Netherlands.

3 Department of Pharmacology, School of Medicine, Faculty of Health Sciences, Aristotle University of Thessaloniki, Thessaloniki, Greece.

4 Unit of Clinical Pharmacology, Department of Internal Medicine, University Hospital Centre Zagreb and University of Zagreb School of Medicine, Zagreb, Croatia

5 School of Health Science, Universidad de La Laguna, Tenerife, Spain.

6 Department of Clinical Pharmacology, Ghent University, Ghent, Belgium.

7 Department of Pharmacology and Clinical Pharmacology, University of Lisbon, Lisbon, Portugal

8 Pharmacology Unit, Department of Medical and Surgical Sciences, Alma Mater Studiorum, University of Bologna

9 Department of Medical and Health Sciences, Linköping University, Linköping, Sweden.

For peer review only

10 Department of Pharmacology-Toxicology, Radboud University Medical Center, Nijmegen, The Netherlands.

11 Health Technologies and Pharmaceuticals Programme, WHO Regional Office for Europe, WHO, Copenhagen, Denmark.

12 Department of Translational Neuroscience, UMC Utrecht Brain Center, University Medical Center Utrecht, Utrecht University, Utrecht, The Netherlands

Word count: 1414

For peer review only

Abstract

Introduction

Junior doctors are responsible for a substantial number of prescribing errors, and final-year medical students lack sufficient prescribing knowledge and skills just before they graduate. Various national and international projects have been initiated to reform the teaching of clinical pharmacology and therapeutics (CP&T) during undergraduate medical training. However, there is as yet no list of commonly prescribed and available medicines that European doctors should be able to independently prescribe safely and effectively without direct supervision. Such a list could form the basis for a European Prescribing Exam and would harmonize European CP&T education. Therefore, the aim of this study is to reach consensus on a list of widely prescribed medicines, available in most EU countries, that European junior doctors should be able to independently prescribe safely and effectively without direct supervision: *The European List of Essential Medicines for Medical Education.*

Methods and analysis

This modified Delphi study will recruit European CP&T teachers (expert group). Two Delphi rounds will be carried out to enable a list to be drawn up of medicines that are available in ≥80% of European countries, which are considered standard prescribing practice, and which junior doctors should be able to prescribe safely and effectively without supervision.

Ethics and dissemination

The study has been approved by the Medical Ethics Review Committee of VU University Medical Center (no. 2020.335) and by the Ethical Review Board of the Netherlands Association for Medical Education (Approved Project no. NVMO-ERB 2020.4.8). The European List of Medicines for Medical Education will be presented at national and international conferences and will be submitted to international peer-reviewed journals. It will also be used to develop and implement the European Prescribing Exam.

For peer review only

Strengths and weaknesses of this study

- To our knowledge, this will be the first study to reach consensus on a European list of essential medicines for medical education.
- The Delphi method is the most suitable method to reach consensus anonymously with a large group of experts working in different countries.
- The *European List of Essential Medicines for Medical Education* will help to harmonize CP&T education in Europe.
- There is already strong collaboration between EACPT and WHO Europe, and the members are recognized experts in the field of CP&T education
- Recruiting a sufficient number of participants from all European countries will be a challenge.

For peer review only

Introduction

Prescribing is a core task of junior doctors, who are responsible for most hospital prescriptions.[1 2] With an ever-expanding therapeutic arsenal and an increasing number of patients with comorbidity and polypharmacy, safe and effective prescribing has become an increasingly complex task. International studies have shown that junior doctors are responsible for a substantial number of prescribing errors [1 3] and that at the time of graduation, junior doctors not only feel insufficiently prepared to prescribe safely and effectively but also have insufficient knowledge and skills to perform this task.[4-8]

This has prompted various national and international projects to reform teaching in clinical pharmacology and therapeutics (CP&T) in the undergraduate medical curriculum. For example, in the United Kingdom and the Netherlands a prescribing assessment has been introduced for final-year students, to ensure that they have acquired the necessary knowledge before graduation.[9-11] Other European countries might benefit from a similar initiative. As many countries do not have the time and resources to implement an assessment at a national level, we initiated an Erasmus+ project (2019-1 - NL01 - KA203-060492) to develop, test, and implement a standardized prescribing assessment on safe prescribing (including knowledge and skills) for undergraduate medical students studying at medical schools in the European Union (i.e. European Prescribing Exam (EuroPE^{+})). For more information, see http://www.prescribingeducation.eu/.

The assessment will be based on the 'essential diseases in prescribing' derived from a Delphi consensus study held in 2018.[12] However, there is no consensus list of medicines that European junior doctors should be able to independently prescribe safely and effectively without direct supervision. This list will form the basis of the European Prescribing Assessment (especially the skills part), and together with country specific adjustments to reform educational programs in CP&T in all European countries. This will complement the wish of the European Association for Clinical Pharmacology and Therapeutics (EACPT) to harmonize European training in CP&T[13] and will be included in a future revision of the Guide to Good Prescribing of the World Health Organisation.[14]

For peer review only - http://bmjopen.bmj.com/site/about/guidelines.xhtml

Previous studies of such lists have been based on the opinions of individuals or small groups of experts or were specific to one country.[15-18] Therefore, the aim of this study is to reach consensus on a list of medicines that are widely prescribed and available in Europe and which European junior doctors should be able to independently prescribe safely and effectively without direct supervision, i.e. *The European List of Essential Medicines for Medical Education.*

Methods and analysis

A modified Delphi method will be used as it has been shown to be an effective and successful method for reaching consensus on content of a CP&T curriculum.[12 19-23] As the availability of medicines differs between European countries, it is not possible to develop an all-encompassing list, but one that can be considered as a basis for European CP&T education. Subsequently, each country can adjust the list based on the availability of medicines in its country. The study will start in October 2020 and will comprise three phases: Phase 1, creating a drug list; selecting an European expert panel; developing a web-based questionnaire in Castor EDC; Phase 2, sending a questionnaire to appointed coordinators; Phase 3, Delphi consensus.

<u>Patient and Public Involvement</u>

No patient involved.

<u>Phase 1</u>

<u>Expert panel</u>

Through the Education Working Group of the European Association for Clinical Pharmacology and Therapeutics (EACPT) and the affiliated Network of Teachers in Pharmacotherapy (NOTIP), all coordinators (n= 393) from all European medical schools (n=297) who are responsible for teaching CP&T to medical students will be approached to participate in the study. The coordinators will be asked to participate in the study themselves and to select a group of experts within their own centre, using the following criteria:

For peer review only

- Two experienced (≥3 years of teaching experience) teachers explicitly engaged in CP&T education for medical students, of which at least one teacher is registered as a clinical pharmacologist;
- At least five medical specialists, preferably a surgeon (e.g. general surgeon); an internist (e.g. general internist, gastroenterologists, pulmonologist, cardiologist); a general practitioner; a specialist in geriatric medicine or geriatrician;
- Two recently graduated junior doctors (graduated ≤1 year ago) who prescribe drugs on a daily basis.

There will be no restrictions regarding the work environment of the respondents (academic or community hospitals). The principal investigator will invite the experts to participate via e-mail, providing an information letter and a link to the online survey. Prior to participation, the experts will be asked to sign a digital informed consent form. In total, we expect to recruit 200-250 experts.

<u>Drug list</u>

On the basis of the WHO Model List of Essential Medicines,[24] guideline therapies for the 'essential diseases in prescribing' (see appendix 1), a literature review, [15-18] and a drug list from the EuroPE$^+$ project,[25] a questionnaire will be developed regarding an extensive list of possible medicines that European junior doctors should be able to prescribe safely and effectively without supervision directly after graduation (appendix 2). This list will be categorized into diseases. For each drug, the most commonly used routes of administration will be listed separately.

<u>Phases 2 and 3</u>

<u>Study design and data collection</u>

In phase 2, the coordinator(s) of each university will receive the list of medicines developed in phase 1 and will be asked to indicate which medicines are available in his or her country. On the basis of this information, a second questionnaire will be drawn up consisting of the medicines that are available in Europe. This questionnaire will be used for phase 3, a two-round Delphi study. In Round 1, all experts will be asked to evaluate two statements for each medicine (item): 1. '*In my country, it*

For peer review only

is standard practice to prescribe this medicine to patients'; 2. 'A junior doctor should be able to independently prescribe this medicine safely and effectively without direct supervision'. Respondents will score both statements using a 5-point Likert scale (1 = strongly disagree; 2 = disagree; 3 = neither agree nor disagree; 4 = agree; 5 = strongly agree). Respondents will also be able to add missing medicines that they consider should be included in Round 2, and to add arguments for their choices in an open text box.

The questionnaire for Round 2 will have the same structure as in Round 1, but will also include the average group score per medicine from Round 1, the suggested medicines, and the arguments for their inclusion. The coordinator will be asked an additional question about the availability of the suggested medicines in his or her country: '*In my country, this medicine is available to prescribe to patients*'.

Statistics

A medicine will be included in the second questionnaire if that medicine is available in ≥80% of the European countries. In accordance with previous studies,[12 22] a medicine will be included in the final European List of Essential Medicines for Medical Education if both statements about this medicine are scored 4 or 5 by ≥80% of the respondents. If one statement about a medicine is scored 4 or 5 by ≥80% of the respondents but the other statement is scored 4 or 5 by ≥ 50% - <80% the respondents, then this medicine will be reassessed in Delphi Round 2. This also applies to suggested medicines. Medicines from Round 2 will be included in the European List of Essential Medicines for Medical Education if both statements regarding a medicine are given a score of 4 or 5 by ≥80% of the respondents. Medicines suggested by respondents should also be available in ≥80% of European countries.

Ethics and dissemination

Prior to participation, all experts will be asked to give their informed consent and provide the following information: email address, medical school, profession with background, and years of clinical and teaching experience. The data will be coded and stored for a maximum of 10 years in a

For peer review only

secure folder on the hard disk of the Amsterdam UMC, location VUmc. Participation will not be professionally advantageous or disadvantageous and there will be no compensation for participation. Respondents can end their participation at any time, without giving a reason. The study has been approved by the Medical Ethics Review Committee of Amsterdam UMC, location VUmc (no. 2020.335) and by the Ethical Review Board of the Netherlands Association for Medical Education (Approved Project no. NVMO-ERB 2020.4.8).

The results of the study will be presented at national and international conferences and will be submitted to international peer-reviewed journals. The final European List of Essential Medicines for Medical Education will be used to develop and implement the European Prescribing Exam.

References

1. Dornan T., Ashcroft D., Heathfield H., et al. An in depth investigation into causes of prescribing errors by foundation trainees in relation to their medical education. EQUIP study. Available at: http://www.gmc-uk.org/FINAL_Report_prevalence_and_causes_of_prescribing_errors.pdf_28935150.pdf., December 2009.
2. Ryan C, Ross S, Davey P, et al. Prevalence and causes of prescribing errors: the PRescribing Outcomes for Trainee Doctors Engaged in Clinical Training (PROTECT) study. *PLoS One* 2014;9(1):e79802. doi: 10.1371/journal.pone.0079802
3. Lewis PJ, Dornan T, Taylor D, et al. Prevalence, incidence and nature of prescribing errors in hospital inpatients: a systematic review. *Drug Saf* 2009;32(5):379-89. doi: 10.2165/00002018-200932050-00002 [published Online First: 2009/05/08]
4. Han WH, Maxwell SR. Are medical students adequately trained to prescribe at the point of graduation? Views of first year foundation doctors. *Scott Med J* 2006;51(4):27-32. doi: 10.1258/RSMSMJ.51.4.27 [published Online First: 2006/12/02]
5. Heaton A, Webb DJ, Maxwell SR. Undergraduate preparation for prescribing: the views of 2413 UK medical students and recent graduates. *British journal of clinical pharmacology* 2008;66(1):128-34. doi: 10.1111/j.1365-2125.2008.03197.x [published Online First: 2008/05/22]
6. Rothwell C, Burford B, Morrison J, et al. Junior doctors prescribing: enhancing their learning in practice. *British journal of clinical pharmacology* 2012;73(2):194-202. doi: 10.1111/j.1365-2125.2011.04061.x [published Online First: 2011/07/15]
7. Brinkman DJ, Tichelaar J, Schutte T, et al. Essential competencies in prescribing: A first european cross-sectional study among 895 final-year medical students. *Clinical pharmacology and therapeutics* 2017;101(2):281-89. doi: 10.1002/cpt.521 [published Online First: 2016/09/21]
8. Brinkman DJ, Tichelaar J, Graaf S, et al. Do final-year medical students have sufficient prescribing competencies? A systematic literature review. *British journal of clinical pharmacology* 2018;84(4):615-35. doi: 10.1111/bcp.13491
9. Kramers C, Janssen BJ, Knol W, et al. A Licence to Prescribe. *British journal of clinical pharmacology* 2017;83(8):1860-61. doi: 10.1111/bcp.13257

For peer review only

10. Jansen DRM, Keijsers C, Kornelissen MO, et al. Towards a "prescribing license" for medical students: development and quality evaluation of an assessment for safe prescribing. *Eur J Clin Pharmacol* 2019 doi: 10.1007/s00228-019-02686-1 [published Online First: 2019/05/20]
11. Maxwell SRJ, Cameron IT, Webb DJ. Prescribing safety: ensuring that new graduates are prepared. *Lancet* 2015;385(9968):579-81. doi: 10.1016/S0140-6736(14)62339-4 [published Online First: 2015/02/24]
12. Jansen BHE, Disselhorst GW, Schutte T, et al. Essential diseases in prescribing: A national Delphi study towards a core curriculum in pharmacotherapy education. *British journal of clinical pharmacology* 2018;84(11):2645-50. doi: 10.1111/bcp.13730 [published Online First: 2018/08/05]
13. Brinkman DJ, Tichelaar J, Okorie M, et al. Pharmacology and Therapeutics Education in the European Union Needs Harmonization and Modernization: A Cross-sectional Survey Among 185 Medical Schools in 27 Countries. *Clinical pharmacology and therapeutics* 2017;102(5):815-22. doi: 10.1002/cpt.682 [published Online First: 2017/03/16]
14. Tichelaar J, Richir MC, Garner S, et al. WHO guide to good prescribing is 25 years old: quo vadis? *European Journal of Clinical Pharmacology* 2020;76(4):507-13. doi: 10.1007/s00228-019-02823-w
15. Audi S, Burrage DR, Lonsdale DO, et al. The 'top 100' drugs and classes in England: an updated 'starter formulary' for trainee prescribers. *British journal of clinical pharmacology* 2018;84(11):2562-71. doi: 10.1111/bcp.13709 [published Online First: 2018/07/06]
16. Baker E, Roberts AP, Wilde K, et al. Development of a core drug list towards improving prescribing education and reducing errors in the UK. *British journal of clinical pharmacology* 2011;71(2):190-8. doi: 10.1111/j.1365-2125.2010.03823.x [published Online First: 2011/01/12]
17. Ross S, Maxwell S. Prescribing and the core curriculum for tomorrow's doctors: BPS curriculum in clinical pharmacology and prescribing for medical students. *British journal of clinical pharmacology* 2012;74(4):644-61. doi: 10.1111/j.1365-2125.2012.04186.x [published Online First: 2012/02/01]
18. Orme M, Frolich J, Vrhovac B, et al. Towards a core curriculum in clinical pharmacology for undergraduate medical students in Europe. *Eur J Clin Pharmacol* 2002;58(9):635-40. doi: 10.1007/s00228-002-0531-9 [published Online First: 2002/12/17]
19. Walley T, Webb DJ. Developing a core curriculum in clinical pharmacology and therapeutics: a Delphi study. *British journal of clinical pharmacology* 1997;44(2):167-70. doi: 10.1046/j.1365-2125.1997.00669.x [published Online First: 1997/08/01]
20. Ross S, Loke YK. Development of learning outcomes for an undergraduate prescribing curriculum (British Pharmacological Society prescribing initiative). *British journal of clinical pharmacology* 2010;70(4):604-8. doi: 10.1111/j.1365-2125.2009.03581.x [published Online First: 2010/09/16]
21. Midlov P, Hoglund P, Eriksson T, et al. Developing a Competency-based Curriculum in Basic and Clinical Pharmacology--A Delphi Study among Physicians. *Basic Clin Pharmacol Toxicol* 2015;117(6):413-20. doi: 10.1111/bcpt.12436 [published Online First: 2015/07/01]
22. Brinkman DJ, Tichelaar J, Mokkink LB, et al. Key Learning Outcomes for Clinical Pharmacology and Therapeutics Education in Europe: A Modified Delphi Study. *Clinical pharmacology and therapeutics* 2018;104(2):317-25. doi: 10.1002/cpt.962 [published Online First: 2017/12/06]
23. Brinkman D, Disselhorst G, Jansen B, et al. What Should Junior Doctors Know about the Drugs they Frequently Prescribe? A Delphi Study among Physicians in the Netherlands. *Basic Clin Pharmacol Toxicol* 2016;118(6):456-61. doi: 10.1111/bcpt.12508 [published Online First: 2015/10/28]
24. Organization WH. World Health Organization Model List of Essential Medicines, 21st List. Geneva, 2019.
25. European Prescribing Exam 2020 [Available from: http://www.prescribingeducation.eu/european-prescribing-exam/.

For peer review only

Authors' contributions

ED, DB, MR and JT were involved in devising the study design and writing the protocol.

TC, YB, FD, RL, KK, JC, ES, PP, MvA MG, RP and SG have reviewed and approved the manuscript.

Funding statement

This work was supported by Erasmus+, grant number 2019-1 - NL01 - KA203-060492

For peer review only

Appendix 1

All guideline medicines to treat the 'essential diseases', ordered alphabetically. When note down more than once, means that there is more than one indication.

Acenocoumarol
Acenocoumarol
Acetylsalicylic acid
Acetylsalysilic acid
Alendronic acid
Aluminium acetotartrate -eardrip
Aluminum hydroxide/magnesium hydroxide
Amitriptyline
Amlodipine
Amoxicillin
Amoxicillin
Amoxicillin
Amoxicillin
Amoxicillin/clavulanate
Amoxicillin/clavulanate
Amoxicillin/clavulanate
Atorvastatin
Azithromycin
Azithromycin
Azithromycin
Azithromycin
Azithromycin
Beclometasone
Betamethasone
Butylscopolamine
Carbasalate calcium
Chloramphenicol
Ciprofloxacin
Citalopram
Clarithromycin
Clarithromycin
Claritromycin
Clemastine
Clemastine
Clomipramine
Clopidogrel
Clopidogrel
Clotrimazole
Codeine
Colchicine
Colecalciferol (with/without) calcium
Desloratadine
Desloratadine
Desloratadine
Desogestrel (oral)
Dexamethason
Dexamethason
Diazepam
Diclofenac
Diclofenac
Diclofenac
Diclofenac
Digoxin
Diltiazem
Diltiazem
Dipyridamole
Domperidone
Domperidone
Doxycycline
Doxycycline
Doxycycline
Doxycycline
Emollients (e.g. Cremor vaselini cetomacrogolis)
Emollients (e.g. Cremor vaselini cetomacrogolis)
Emollients (e.g. Cremor vaselini cetomacrogolis)
Enalapril
Epinephrine (adrenaline)
Epinephrine (adrenaline)
Esomeprazole
Ethinylestradiol/levonorgestrel (oral)
Etonogestrel (s.c. Implant)
Ferrous fumarate
Flucloxacillin
Flucloxacillin
Flucloxacillin
Fluconazole
Fluticasone
Fluticasone
Folic acid (vitamin b9)
Formoterol
Fosfomycin
Fusidic acid
Gliclazide
Glucagon
Glucose solution
Glyceryl trinitrate (nitroglycerine)
Haloperidol
Hydrochlorothiazide
Hydrocortisone
Hydrocortisone
Hydrocortisone/acetic acid

For peer review only

Hydroxocobalamin (vitamin b12)	Omeprazole, pantoprazole, esomeprazole)
Ibuprofen	Ors (oral rehydration solution)
Ibuprofen	Ors (oral rehydration solution)
Ibuprofen	Oxazepam
Ibuprofen	Oxycodone
Influenza vaccine	Pantoprazole
Insulin aspart	Paracetamol
Insulin glargine	Paracetamol
Ipratropium bromide	Paroxetine
Ipratropium bromide	Perindopril
Isosorbide dinitrate	Pheneticillin
Isosorbide mononitrate	Pheneticillin
Ispaghula (psylla seeds)	Pheneticillin
Lactulose	Phenprocoumon
Levocabastine	Phenprocoumon
Levocetirizine	Prednisolone
Levocetirizine	Prednisolone
Levocetirizine	Prednisolone
Levonorgestrel (iud)	Prensisolone
Levothyroxine	Propranolol
Lidocaine creme	Ranitidine
Lisinopril	Risedronic acid
Loperamide	Rosuvastatin
Loperamide	Salbutamol
Lorazepam	Salbutamol
Losartan	Salbutamol
Macrogol	Salbutamol
Macrogol	Salbutamol
Mebeverine	Salbutamol
Medroxyprogesterone (s.c. Depot)	Salmeterol
Menthol in aqueous cream	Simvastatin
Menthol in aqueous cream	Sodium chloride solution
Metformin	Spironolactone
Metocloperamide	Temazepam
Metocloperamide	Tetanus toxoid
Metoprolol	Thiamine (vit b1)
Metoprolol	Tiotropium bromide
Metronidazole	Tramadol
Miconazol	Triamcinolone
Midazolam (i.m.)	Triamcinolone/acetic acid
Mometasone	Trimethoprim
Nadroparin	Trimethoprim/polymyxine b
Nadroparin	Trimethoprim/sulfamethoxazole
Naproxen	Trimethoprim/sulfamethoxazole
Naproxen	Trimethoprim/sulfamethoxazole
Naproxen	Verapamil
Naproxen	Verapamil
Nitrofurantoin	Xylometazoline nasalspray
Omeprazole	Xylometazoline nasalspray
Omeprazole, pantoprazole	Zolpidem

For peer review only

List of medicines	Route of administration	Route of administration	Route of administration	Route of administration
Miconazole	Oral			
Aluminium hydroxide/magnesium hydroxide	Oral			
Magnesium hydroxide	Oral			
Ranitidine	Oral			
Misoprostol	Oral			
Omeprazole	Oral			
Pantoprazole	Oral			
Esomeprazole	Oral	IV		
Mebeverine	Oral			
Atropine	IV			
Butylscopolamine	Oral			
Metoclopramide	Oral	IV	Rectal	
Domperidone	Oral			
Ondansetron	Oral	IV	Rectal	
Bisacodyl	Oral	Rectal		
Ispaghula husk	Oral			
Lactulose	Oral			
Macrogol	Oral			
Nystatin	Oral			
Oral rehydration solution (ORS)	Oral			
Loperamide	Oral			
Insulin	SC			
Insulin aspart	SC			
Protamine (novomix)	SC			
Insulin glargine	SC			
Metformin	Oral			
Gliclazide	Oral			
Glimepiride	Oral			
Acarbose	Oral			
Pioglitazone	Oral			
Sitagliptin	Oral			
Linagliptin	Oral			
Exenatide	SC			
Liraglutide	SC			
Canagliflozin	Oral			
Empagliflozin	Oral			
Repaglinide	Oral			
Cholecalciferol	Oral			
Thiamine (vit B1)	Oral	IV		
Calcium/vitamin D	Oral			
Potassium chloride	Oral			
Fenprocoumon	SC			
Acenocoumarol	SC			
Heparin	IV			

For peer review only

Enoxaparin	SC	
Nadroparin	SC	
Clopidogrel	Oral	
Dipyridamole	Oral	
Carbasalate calcium	Oral	
Prasugrel	Oral	
Dabigatran	Oral	
Rivaroxaban	Oral	
Apixaban	Oral	
Fondaparinux	SC	IV
Tranexamic acid	Oral	IV
Vitamin K	Oral	IV
Prothrombin complex concentrate	IV	
Ferrous fumarate	Oral	
Hydroxocobalamin (vitamin B12)	SC	IM
Folium acid (vitamin B9)	Oral	
Glucose solution	IV	
Potassium chloride	IV	
Digoxin	Oral	IV
Flecainide	Oral	
Amiodarone	Oral	IV
Epinephrine (adrenaline)	IM	
Nitroglycerine	SL	
Isosorbide dinitrate	Oral	SL
Isosorbide mononitrate	Oral	
Methyldopa	Oral	
Hydrochlorothiazide	Oral	
Chlorthalidone	Oral	
Furosemide	Oral	IV
Bumetanide	Oral	IV
Spironolactone	Oral	
Propranolol	Oral	
Sotalol	Oral	
Metoprolol	Oral	IV
Atenolol	Oral	
Bisoprolol	Oral	
Labetolol	Oral	IV
Amlodipine	Oral	
Nifedipine	Oral	
Barnidipine	Oral	
Lercanidipine	Oral	
Nicardipine	IV	
Verapamil	Oral	IV
Diltiazem	Oral	
Enalapril	Oral	
Lisinopril	Oral	

For peer review only

Perindopril	Oral	
Ramipril	Oral	
Fosinopril	Oral	
Losartan	Oral	
Valsartan	Oral	
Irbesartan	Oral	
Candesartan	Oral	
Simvastatin	Oral	
Pravastatin	Oral	
Atorvastatin	Oral	
Rosuvastatin	Oral	
Gemfibrozil	Oral	
Colestyramine	Oral	
Ezetimibe	Oral	
Evolocumab	SC	
Miconazole	Cutaneous	
Emollients (e.g. Cremor vaseline cetomacrogolis)	Cutaneous	
Zink oxide	Cutaneous	
Vaseline and other	Cutaneous	
Lidocaine creme	Cutaneous	
Menthol in hydrophilic ointment	Cutaneous	
Fusidic acid	Cutaneous	
Hydrocortisone	Cutaneous	
Triamcinolone	Cutaneous	
Betamethasone	Cutaneous	
Mometasone	Cutaneous	
Clobetasole	Cutaneous	
Clotrimazole	Cutaneous	
Miconazole	Cutaneous	
IUD with progestogen	IUD	
Ethinylestradiol/levonorgestrel	Oral	
Medroxyprogesterone (s.c. Depot)	SC	
Etonogestrel (s.c. Implant)	SC	
Desogestrel	Oral	
Levonorgestrel	Oral	
Estradiol	Oral	
Oxybutynin	Oral	
Solifenacin	Oral	
Tamsulosin	Oral	
Finasteride	Oral	
Betamethasone	Oral	IV
Dexamethasone	Oral	IV
Prednisolone	Oral	IV
Hydrocortisone	Oral	IV
Levothyroxine	Oral	

For peer review only

Glucagon	IM			
Doxycycline	Oral			
Amoxicillin	Oral	IV		
Feneticillin	Oral	IV		
Flucloxacillin	Oral	IV		
Amoxicillin/clavulanic acid	Oral	IV		
Meropenem	IV			
Trimethoprim	Oral			
Co-trimoxazole	Oral	IV		
Trimethoprim/sulfametoxazole	Oral	IV		
Erythromycin	Oral	IV		
Clarithromycin	Oral	IV		
Azithromycin	Oral	IV		
Gentamycin	IV			
Ciprofloxacin	Oral	IV		
Vancomycin	IV			
Fusidic acid	Oral			
Nitrofurantoin	Oral			
Fosfomycin	Oral			
Fluconazole	Oral	IV		
Rifampicin	Oral	IV		
Acyclovir	Oral	IV		
Valaciclovir	Oral			
Oseltamivir	Oral			
Tetanus toxoid	IM			
Methotrexate	Oral	IV	IM	SC
Diclofenac	Oral			
Ibuprofen	Oral			
Naproxen	Oral			
Ibuprofen	Cutaneous			
Diclofenac	Cutaneous			
Allopurinol	Oral			
Colchicine	Oral			
Alendronic acid	Oral			
Risedronic acid	Oral			
Denosumab	SC			
Fentanyl	IV			
Lidocaine	SC			
Epinephrine (adrenaline)	SC			
Morphine	Oral	IV	SC	
Oxycodone	Oral	SC		
Fentanyl	Oral	Cutaneous	Nasal	SL
Tramadol	Oral	Rectal		
Piritramide	SC			
Acetylsalicylic acid	Oral	IV		
Calcium carbasalate	Oral	IV		

For peer review only

Paracetamol	Oral	IV	Rectal
Sumatriptan	Oral	Nasal	SC
Valproic acid	Oral	IV	
Levetiracetam	Oral	IV	
Pregabalin	Oral	IV	
Haloperidol	Oral	IV	
Clozapine	Oral		
Olanzapine	Oral		
Quetiapine	Oral		
Lithium	Oral		
Risperidone	Oral	IM	
Diazepam	Oral	Rectal	
Oxazepam	Oral		
Lorazepam	Oral	IM	IV
Temazepam	Oral		
Midazolam	Oral	IV	
Zolpidem	Oral		
Clomipramine	Oral		
Amitriptyline	Oral		
Nortriptyline	Oral		
Fluoxetine	Oral		
Citalopram	Oral		
Paroxetine	Oral		
Sertraline	Oral		
Escitalopram	Oral		
Mirtazapine	Oral		
Venlafaxine	Oral		
Metronidazole	Oral	IV	
Xylometazoline	Nasal		
Levocabastine	Ocular		
Beclomethasone	Nasal		
Budesonide	Nasal		
Mometasone	Nasal		
Fluticasone	Nasal		
Salbutamol	Inhalation		
Salmeterol	Inhalation		
Formoterol	Inhalation		
Beclometasone	Inhalation		
Budesonide	Inhalation		
Fluticasone	Inhalation		
Ciclesonide	Inhalation		
Ipratropium bromide	Inhalation		
Tiotropium bromide	Inhalation		
Tiotropium	Inhalation		
Codeine	Oral		
Clemastine	Oral	IV	

For peer review only

Meclozine	Oral	
Levocetirizine	Oral	
Desloratadine	Oral	
Chloramphenicol	Ocular	
Fusidic acid	Ocular	
Erythromycin	Ocular	
Trimethoprim/polymyxin B	Ocular	
Dexamethasone (Ocular)	Ocular	
Prednisolone (Ocular)	Ocular	
Levocabastine	Ocular	
Aluminium acetate	Ear	
Miconazole	Oral	
Hydrocortisone/acetic acid	Ear	
Triamcinolone/acetic acid	Ear	
Protamine	IV	
Naloxone	IV	
Flumazenil	IV	
Ticagrelor	Oral	IV
Cefuroxime	IV	
Cefotaxime	IV	
Ceftriaxone	IV	
Ceftazidime	IV	
Clindamycin	Oral	IV
Sodium chloride	IV	
Ringer's lactate	IV	

For peer review only

BMJ Open

The European List of Essential Medicines for Medical Education, a Protocol for a Modified Delphi Study

Journal:	*BMJ Open*
Manuscript ID	bmjopen-2020-045635.R1
Article Type:	Protocol
Date Submitted by the Author:	03-Mar-2021
Complete List of Authors:	Donker, Erik; Amsterdam UMC Locatie VUmc, Department of Internal Medicine, section Pharmacotherapy; Research and Expertise Centre in Pharmacotherapy Education (RECIPE)<br>Brinkman, David; Amsterdam UMC Locatie VUmc, Department of Internal Medicine, section Pharmacotherapy; Research and Expertise Centre in Pharmacotherapy Education (RECIPE)<br>Richir, Milan; Amsterdam UMC Locatie VUmc, Department of Internal Medicine, section Pharmacotherapy; Research and Expertise Centre in Pharmacotherapy Education (RECIPE)<br>Papaioannidou, Paraskevi; Aristotle University of Thessaloniki Faculty of Health Sciences, Department of Pharmacology, School of Medicine<br>Likic, Robert; University of Zagreb School of Medicine; University Hospital Centre Zagreb, Department of Internal Medicine, Unit of Clinical Pharmacology<br>Sanz, Emilio ; Universidad de La Laguna, School of Health Science<br>Christiaens, Thierry; Ghent University, Department of Clinical Pharmacology<br>Costa, Joao; University of Lisbon, Department of Pharmacology and Clinical Pharmacology<br>De Ponti, Fabrizio; University of Bologna, Department of Medical and Surgical Sciences, Pharmacology Unit<br>Gatti, Milo; University of Bologna, Department of Medical and Surgical Sciences, Pharmacology Unit<br>Böttiger, Ylva; Linkopings universitet, Department of Medical and Health Sciences<br>Kramers, Cornelis; Radboud University Medical Center, Department of Internal Medicine and Pharmacology-Toxicology<br>Garners, Sarah; WHO Regional Office for Europe, Health Technologies and Pharmaceuticals Programme<br>Pandit, Rahul; University Medical Centre Utrecht Brain Centre, Department of Translational Neuroscience<br>van Agtmael, Michiel; Amsterdam UMC Locatie VUmc, Department of Internal Medicine, section Pharmacotherapy; Research and Expertise Centre in Pharmacotherapy Education (RECIPE)<br>Tichelaar, Jelle; Amsterdam UMC Locatie VUmc, Department of Internal Medicine, section Pharmacotherapy; Research and Expertise Centre in Pharmacotherapy Education (RECIPE)
**Primary Subject Heading**:	Medical education and training

For peer review only - http://bmjopen.bmj.com/site/about/guidelines.xhtml

Secondary Subject Heading:	Pharmacology and therapeutics
Keywords:	EDUCATION & TRAINING (see Medical Education & Training), MEDICAL EDUCATION & TRAINING, THERAPEUTICS

SCHOLARONE™ Manuscripts

I, the Submitting Author has the right to grant and does grant on behalf of all authors of the Work (as defined in the below author licence), an exclusive licence and/or a non-exclusive licence for contributions from authors who are: i) UK Crown employees; ii) where BMJ has agreed a CC-BY licence shall apply, and/or iii) in accordance with the terms applicable for US Federal Government officers or employees acting as part of their official duties; on a worldwide, perpetual, irrevocable, royalty-free basis to BMJ Publishing Group Ltd ("BMJ") its licensees and where the relevant Journal is co-owned by BMJ to the co-owners of the Journal, to publish the Work in this journal and any other BMJ products and to exploit all rights, as set out in our licence.

The Submitting Author accepts and understands that any supply made under these terms is made by BMJ to the Submitting Author unless you are acting as an employee on behalf of your employer or a postgraduate student of an affiliated institution which is paying any applicable article publishing charge ("APC") for Open Access articles. Where the Submitting Author wishes to make the Work available on an Open Access basis (and intends to pay the relevant APC), the terms of reuse of such Open Access shall be governed by a Creative Commons licence – details of these licences and which Creative Commons licence will apply to this Work are set out in our licence referred to above.

Other than as permitted in any relevant BMJ Author's Self Archiving Policies, I confirm this Work has not been accepted for publication elsewhere, is not being considered for publication elsewhere and does not duplicate material already published. I confirm all authors consent to publication of this Work and authorise the granting of this licence.

For peer review only

The European List of Essential Medicines for Medical Education, a Protocol for a Modified Delphi Study

Corresponding and principle author
Erik (Martin) Donker
Amsterdam UMC, location VUmc
Dept. Internal medicine, Section Pharmacotherapy
De Boelelaan 1117
1081HV Amsterdam
The Netherlands.
e.donker@amsterdamumc.nl

Co-authors and affiliations
David J. Brinkman[1,2]
Milan C. Richir[1,2]
Paraskevi Papaioannidou[3]
Robert Likic[4]
Emilio J Sanz[5]
Thierry Christiaens[6]
João N Costa[7]
Fabrizio De Ponti[8]
Milo Gatti[8]
Ylva Böttiger[9]
Cornelis Kramers[10]
Sarah Garner[11]
Rahul Padit[12]
Michiel A van Agtmael[1,2]
Jelle Tichelaar[1,2]

1 Department of Internal Medicine, section Pharmacotherapy, Amsterdam UMC, location VUmc, Amsterdam, Netherlands

2 Research and Expertise Centre in Pharmacotherapy Education (RECIPE), Amsterdam, The Netherlands.

3 Department of Pharmacology, School of Medicine, Faculty of Health Sciences, Aristotle University of Thessaloniki, Thessaloniki, Greece.

4 Unit of Clinical Pharmacology, Department of Internal Medicine, University Hospital Centre Zagreb and University of Zagreb School of Medicine, Zagreb, Croatia

5 School of Health Science, Universidad de La Laguna, Tenerife, Spain.

6 Department of Clinical Pharmacology, Ghent University, Ghent, Belgium.

7 Department of Pharmacology and Clinical Pharmacology, University of Lisbon, Lisbon, Portugal

8 Pharmacology Unit, Department of Medical and Surgical Sciences, Alma Mater Studiorum, University of Bologna

9 Department of Medical and Health Sciences, Linköping University, Linköping, Sweden.

For peer review only

For peer review only - http://bmjopen.bmj.com/site/about/guidelines.xhtml

10 Department of Pharmacology-Toxicology, Radboud University Medical Center, Nijmegen, The Netherlands.

11 Health Technologies and Pharmaceuticals Programme, WHO Regional Office for Europe, WHO, Copenhagen, Denmark.

12 Department of Translational Neuroscience, UMC Utrecht Brain Center, University Medical Center Utrecht, Utrecht University, Utrecht, The Netherlands

Word count: 1504

For peer review only

For peer review only - http://bmjopen.bmj.com/site/about/guidelines.xhtml

Abstract

Introduction

Junior doctors are responsible for a substantial number of prescribing errors, and final-year medical students lack sufficient prescribing knowledge and skills just before they graduate. Various national and international projects have been initiated to reform the teaching of clinical pharmacology and therapeutics (CP&T) during undergraduate medical training. However, there is as yet no list of commonly prescribed and available medicines that European doctors should be able to independently prescribe safely and effectively without direct supervision. Such a list could form the basis for a European Prescribing Exam and would harmonize European CP&T education. Therefore, the aim of this study is to reach consensus on a list of widely prescribed medicines, available in most EU countries, that European junior doctors should be able to independently prescribe safely and effectively without direct supervision: *The European List of Essential Medicines for Medical Education.*

Methods and analysis

This modified Delphi study will recruit European CP&T teachers (expert group). Two Delphi rounds will be carried out to enable a list to be drawn up of medicines that are available in ≥80% of European countries, which are considered standard prescribing practice, and which junior doctors should be able to prescribe safely and effectively without supervision.

Ethics and dissemination

The study has been approved by the Medical Ethics Review Committee of VU University Medical Center (no. 2020.335) and by the Ethical Review Board of the Netherlands Association for Medical Education (Approved Project no. NVMO-ERB 2020.4.8). The European List of Medicines for Medical Education will be presented at national and international conferences and will be submitted to international peer-reviewed journals. It will also be used to develop and implement the European Prescribing Exam.

For peer review only - http://bmjopen.bmj.com/site/about/guidelines.xhtml

Strengths and weaknesses of this study

- To our knowledge, this will be the first study to reach consensus on a European list of essential medicines for medical education.
- The Delphi method is the most suitable method to reach consensus anonymously with a large group of experts working in different countries.
- The *European List of Essential Medicines for Medical Education* will help to harmonize CP&T education in Europe.
- There is already strong collaboration between EACPT and WHO Europe, and the members are recognized experts in the field of CP&T education
- Recruiting a sufficient number of participants from all European countries will be a challenge.

For peer review only

Introduction

Prescribing is a core task of junior doctors, who are responsible for most hospital prescriptions.[1 2] With an ever-expanding therapeutic arsenal and an increasing number of patients with comorbidity and polypharmacy, safe and effective prescribing has become an increasingly complex task. International studies have shown that junior doctors are responsible for a substantial number of prescribing errors [1 3] and that at the time of graduation, junior doctors not only feel insufficiently prepared to prescribe safely and effectively but also have insufficient knowledge and skills to perform this task.[4-8]

This has prompted various national and international projects to reform teaching in clinical pharmacology and therapeutics (CP&T) in the undergraduate medical curriculum. For example, in the United Kingdom and the Netherlands a prescribing assessment has been introduced for final-year students, to ensure that they have acquired the necessary knowledge before graduation.[9-11] Other European countries might benefit from a similar initiative. As many countries do not have the time and resources to implement an assessment at a national level, we initiated an Erasmus+ project (2019-1 - NL01 - KA203-060492) to develop, test, and implement a standardized prescribing assessment on safe prescribing (including knowledge and skills) for undergraduate medical students studying at medical schools in the European Union (i.e. European Prescribing Exam (EuroPE^{+})). For more information, see http://www.prescribingeducation.eu/.

The assessment will be based on the 'essential diseases in prescribing' derived from a Delphi consensus study held in 2018.[12] However, there is no consensus list of medicines that European junior doctors should be able to independently prescribe safely and effectively without direct supervision. This list will form the basis of the European Prescribing Exam (especially the skills part), and together with country specific adjustments to reform educational programs in CP&T in all European countries. This will complement the wish of the European Association for Clinical Pharmacology and Therapeutics (EACPT) to harmonize European training in CP&T[13] and will be included in a future revision of the Guide to Good Prescribing of the World Health Organisation.[14]

For peer review only - http://bmjopen.bmj.com/site/about/guidelines.xhtml

Previous studies of such lists have been based on the opinions of individuals or small groups of experts or were specific to one country.[15-18] Therefore, the aim of this study is to reach consensus on a list of medicines that are widely prescribed and available in Europe and which European junior doctors should be able to independently prescribe safely and effectively without direct supervision, i.e. *The European List of Essential Medicines for Medical Education.*

Methods and analysis

A modified Delphi method will be used as it has been shown to be an effective and successful method for reaching consensus on content of a CP&T curriculum.[12 19-23] As the availability of medicines differs between European countries, it is not possible to develop an all-encompassing list, but one that can be considered as a basis for European CP&T education. Subsequently, each country can adjust the list based on the availability of medicines in its country. The study will start in October 2020 and will comprise three phases: Phase 1, creating a drug list; selecting an European expert panel; developing a web-based questionnaire in Castor EDC; Phase 2, sending a questionnaire to appointed coordinators; Phase 3, Delphi consensus.

Patient and Public Involvement

No patient involved.

Phase 1

Drug list

On the basis of the WHO Model List of Essential Medicines,[24] guideline therapies for the 'essential diseases in prescribing' (see appendix 1), a literature review,[15-18] and a drug list from the EuroPE$^+$ project,[25] a questionnaire will be developed regarding an extensive list of possible medicines that European junior doctors should be able to prescribe safely and effectively without supervision directly after graduation (appendix 2). This list will be categorized into diseases. For each drug, the most commonly used routes of administration will be listed separately.

For peer review only - http://bmjopen.bmj.com/site/about/guidelines.xhtml

For peer review only

Expert panel

Through the Education Working Group of the European Association for Clinical Pharmacology and Therapeutics (EACPT) and the affiliated Network of Teachers in Pharmacotherapy (NOTIP), all coordinators (n= 393) from all European medical schools (n=297) who are responsible for teaching CP&T to medical students will be approached to participate in the study. The coordinators will be asked to participate in the study themselves and to select a group of experts within their own centre, using the following criteria:

- Two experienced (≥3 years of teaching experience) teachers explicitly engaged in CP&T education for medical students, of which at least one teacher is registered as a clinical pharmacologist;
- At least five healthcare professionals, preferably a surgeon (e.g. general surgeon); an internist (e.g. general internist, gastroenterologists, pulmonologist, cardiologist); a general practitioner; a specialist in geriatric medicine or geriatrician; a (hospital)pharmacist;
- Two recently graduated junior doctors (graduated ≤1 year ago) who prescribe drugs on a daily basis.

There will be no restrictions regarding the work environment of the respondents (academic or community hospitals). The principal investigator will invite the experts to participate via e-mail, providing an information letter and a link to the online survey. Prior to participation, the experts will be asked to sign a digital informed consent form.

Based on previous studies with this dedicated group of experts,[22 26] we expect a response rate of 25% for the coordinators, representing all European countries. Assuming 5-6 recruited experts per coordinator and a response rate of 25% as well, we expect in total 200-250 experts to complete the study."

Phases 2 and 3

Study design and data collection

For peer review only - http://bmjopen.bmj.com/site/about/guidelines.xhtml

In phase 2, the coordinator(s) of each university will receive the list of medicines developed in phase 1 and will be asked to indicate which medicines are available in his or her country. On the basis of this information, a second questionnaire will be drawn up consisting of the medicines that are available in Europe. This questionnaire will be used for phase 3, a two-round Delphi study. In Round 1, all experts will be asked to evaluate two statements for each medicine (item): 1. '*In my country, it is standard practice to prescribe this medicine to patients*'; *2. 'A junior doctor should be able to independently prescribe this medicine safely and effectively without direct supervision'.* Respondents will score both statements using a 5-point Likert scale (1 = strongly disagree; 2 = disagree; 3 = neither agree nor disagree; 4 = agree; 5 = strongly agree). Respondents will also be able to add missing medicines that they consider should be included in Round 2, and to add arguments for their choices in an open text box.

The questionnaire for Round 2 will have the same structure as in Round 1, but will also include the average group score per medicine from Round 1, the suggested medicines, and the arguments for their inclusion. The coordinator will be asked an additional question about the availability of the suggested medicines in his or her country: '*In my country, this medicine is available to prescribe to patients*'.

In order to minimize participant drop-out, the list of medicines will be structured and participants are allowed to complete a portion of the survey and return later to finish the remaining part. The list must be completed within two weeks, a reminder will be send after one week.

Statistics

A medicine will be included in the second questionnaire if that medicine is available in ≥80% of the European countries. In accordance with previous studies,[12 22] a medicine will be included in the final European List of Essential Medicines for Medical Education if both statements about this medicine are scored 4 or 5 by ≥80% of the respondents. If one statement about a medicine is scored 4 or 5 by ≥80% of the respondents but the other statement is scored 4 or 5 by ≥ 50% - <80% the respondents, then this medicine will be reassessed in Delphi Round 2. This also applies to suggested medicines.

For peer review only - http://bmjopen.bmj.com/site/about/guidelines.xhtml

Medicines from Round 2 will be included in the European List of Essential Medicines for Medical Education if both statements regarding a medicine are given a score of 4 or 5 by ≥80% of the respondents. Medicines suggested by respondents should also be available in ≥80% of European countries.

Ethics and dissemination

Prior to participation, all experts will be asked to give their informed consent and provide the following information: email address, medical school, profession with background, and years of clinical and teaching experience. The data will be coded and stored for a maximum of 10 years in a secure folder on the hard disk of the Amsterdam UMC, location VUmc. Participation will not be professionally advantageous or disadvantageous and there will be no compensation for participation. Respondents can end their participation at any time, without giving a reason. The study has been approved by the Medical Ethics Review Committee of Amsterdam UMC, location VUmc (no. 2020.335) and by the Ethical Review Board of the Netherlands Association for Medical Education (Approved Project no. NVMO-ERB 2020.4.8).

The results of the study will be presented at national and international conferences and will be submitted to international peer-reviewed journals. The final European List of Essential Medicines for Medical Education will be used to develop and implement the European Prescribing Exam.

References

1. Dornan T., Ashcroft D., Heathfield H., et al. An in depth investigation into causes of prescribing errors by foundation trainees in relation to their medical education. EQUIP study. Available at: http://www.gmc-uk.org/FINAL_Report_prevalence_and_causes_of_prescribing_errors.pdf_28935150.pdf., December 2009.
2. Ryan C, Ross S, Davey P, et al. Prevalence and causes of prescribing errors: the PRescribing Outcomes for Trainee Doctors Engaged in Clinical Training (PROTECT) study. *PLoS One* 2014;9(1):e79802. doi: 10.1371/journal.pone.0079802
3. Lewis PJ, Dornan T, Taylor D, et al. Prevalence, incidence and nature of prescribing errors in hospital inpatients: a systematic review. *Drug Saf* 2009;32(5):379-89. doi: 10.2165/00002018-200932050-00002 [published Online First: 2009/05/08]

For peer review only - http://bmjopen.bmj.com/site/about/guidelines.xhtml

4. Han WH, Maxwell SR. Are medical students adequately trained to prescribe at the point of graduation? Views of first year foundation doctors. *Scott Med J* 2006;51(4):27-32. doi: 10.1258/RSMSMJ.51.4.27 [published Online First: 2006/12/02]
5. Heaton A, Webb DJ, Maxwell SR. Undergraduate preparation for prescribing: the views of 2413 UK medical students and recent graduates. *British journal of clinical pharmacology* 2008;66(1):128-34. doi: 10.1111/j.1365-2125.2008.03197.x [published Online First: 2008/05/22]
6. Rothwell C, Burford B, Morrison J, et al. Junior doctors prescribing: enhancing their learning in practice. *British journal of clinical pharmacology* 2012;73(2):194-202. doi: 10.1111/j.1365-2125.2011.04061.x [published Online First: 2011/07/15]
7. Brinkman DJ, Tichelaar J, Schutte T, et al. Essential competencies in prescribing: A first european cross-sectional study among 895 final-year medical students. *Clinical pharmacology and therapeutics* 2017;101(2):281-89. doi: 10.1002/cpt.521 [published Online First: 2016/09/21]
8. Brinkman DJ, Tichelaar J, Graaf S, et al. Do final-year medical students have sufficient prescribing competencies? A systematic literature review. *British journal of clinical pharmacology* 2018;84(4):615-35. doi: 10.1111/bcp.13491
9. Kramers C, Janssen BJ, Knol W, et al. A Licence to Prescribe. *British journal of clinical pharmacology* 2017;83(8):1860-61. doi: 10.1111/bcp.13257
10. Jansen DRM, Keijsers C, Kornelissen MO, et al. Towards a "prescribing license" for medical students: development and quality evaluation of an assessment for safe prescribing. *Eur J Clin Pharmacol* 2019 doi: 10.1007/s00228-019-02686-1 [published Online First: 2019/05/20]
11. Maxwell SRJ, Cameron IT, Webb DJ. Prescribing safety: ensuring that new graduates are prepared. *Lancet* 2015;385(9968):579-81. doi: 10.1016/S0140-6736(14)62339-4 [published Online First: 2015/02/24]
12. Jansen BHE, Disselhorst GW, Schutte T, et al. Essential diseases in prescribing: A national Delphi study towards a core curriculum in pharmacotherapy education. *British journal of clinical pharmacology* 2018;84(11):2645-50. doi: 10.1111/bcp.13730 [published Online First: 2018/08/05]
13. Brinkman DJ, Tichelaar J, Okorie M, et al. Pharmacology and Therapeutics Education in the European Union Needs Harmonization and Modernization: A Cross-sectional Survey Among 185 Medical Schools in 27 Countries. *Clinical pharmacology and therapeutics* 2017;102(5):815-22. doi: 10.1002/cpt.682 [published Online First: 2017/03/16]
14. Tichelaar J, Richir MC, Garner S, et al. WHO guide to good prescribing is 25 years old: quo vadis? *European Journal of Clinical Pharmacology* 2020;76(4):507-13. doi: 10.1007/s00228-019-02823-w
15. Audi S, Burrage DR, Lonsdale DO, et al. The 'top 100' drugs and classes in England: an updated 'starter formulary' for trainee prescribers. *British journal of clinical pharmacology* 2018;84(11):2562-71. doi: 10.1111/bcp.13709 [published Online First: 2018/07/06]
16. Baker E, Roberts AP, Wilde K, et al. Development of a core drug list towards improving prescribing education and reducing errors in the UK. *British journal of clinical pharmacology* 2011;71(2):190-8. doi: 10.1111/j.1365-2125.2010.03823.x [published Online First: 2011/01/12]
17. Ross S, Maxwell S. Prescribing and the core curriculum for tomorrow's doctors: BPS curriculum in clinical pharmacology and prescribing for medical students. *British journal of clinical pharmacology* 2012;74(4):644-61. doi: 10.1111/j.1365-2125.2012.04186.x [published Online First: 2012/02/01]
18. Orme M, Frolich J, Vrhovac B, et al. Towards a core curriculum in clinical pharmacology for undergraduate medical students in Europe. *Eur J Clin Pharmacol* 2002;58(9):635-40. doi: 10.1007/s00228-002-0531-9 [published Online First: 2002/12/17]
19. Walley T, Webb DJ. Developing a core curriculum in clinical pharmacology and therapeutics: a Delphi study. *British journal of clinical pharmacology* 1997;44(2):167-70. doi: 10.1046/j.1365-2125.1997.00669.x [published Online First: 1997/08/01]

For peer review only - http://bmjopen.bmj.com/site/about/guidelines.xhtml

20. Ross S, Loke YK. Development of learning outcomes for an undergraduate prescribing curriculum (British Pharmacological Society prescribing initiative). *British journal of clinical pharmacology* 2010;70(4):604-8. doi: 10.1111/j.1365-2125.2009.03581.x [published Online First: 2010/09/16]
21. Midlov P, Hoglund P, Eriksson T, et al. Developing a Competency-based Curriculum in Basic and Clinical Pharmacology--A Delphi Study among Physicians. *Basic Clin Pharmacol Toxicol* 2015;117(6):413-20. doi: 10.1111/bcpt.12436 [published Online First: 2015/07/01]
22. Brinkman DJ, Tichelaar J, Mokkink LB, et al. Key Learning Outcomes for Clinical Pharmacology and Therapeutics Education in Europe: A Modified Delphi Study. *Clinical pharmacology and therapeutics* 2018;104(2):317-25. doi: 10.1002/cpt.962 [published Online First: 2017/12/06]
23. Brinkman D, Disselhorst G, Jansen B, et al. What Should Junior Doctors Know about the Drugs they Frequently Prescribe? A Delphi Study among Physicians in the Netherlands. *Basic Clin Pharmacol Toxicol* 2016;118(6):456-61. doi: 10.1111/bcpt.12508 [published Online First: 2015/10/28]
24. Organization WH. World Health Organization Model List of Essential Medicines, 21st List. Geneva, 2019.
25. European Prescribing Exam 2020 [Available from: http://www.prescribingeducation.eu/european-prescribing-exam/.
26. Bakkum MJ, Tichelaar J, Papaioannidou P, et al. Harmonizing and improving European education in prescribing: An overview of digital educational resources used in clinical pharmacology and therapeutics. *British journal of clinical pharmacology* 2020 doi: 10.1111/bcp.14453 [published Online First: 2020/07/09]

Authors' contributions

ED, DB, MR and JT were involved in devising the study design and writing the protocol.

TC, YB, FD, RL, CK, JC, ES, PP, MvA, MG, RP and SG have reviewed and approved the manuscript.

Funding statement

This work was funded by Erasmus+, grant number 2019-1 - NL01 - KA203-060492

Competing interests: SG reports her employment at WHO Europe, all other authors declare no

conflict of interest.

For peer review only

Appendix 1

All guideline medicines to treat the 'essential diseases', ordered alphabetically. When note down more than once, means that there is more than one indication.

Acenocoumarol
Acenocoumarol
Acetylsalicylic acid
Acetylsalysilic acid
Alendronic acid
Aluminium acetotartrate -eardrip
Aluminum hydroxide/magnesium hydroxide
Amitriptyline
Amlodipine
Amoxicillin
Amoxicillin
Amoxicillin
Amoxicillin
Amoxicillin/clavulanate
Amoxicillin/clavulanate
Amoxicillin/clavulanate
Atorvastatin
Azithromycin
Azithromycin
Azithromycin
Azithromycin
Azithromycin
Beclometasone
Betamethasone
Butylscopolamine
Carbasalate calcium
Chloramphenicol
Ciprofloxacin
Citalopram
Clarithromycin
Clarithromycin
Claritromycin
Clemastine
Clemastine
Clomipramine
Clopidogrel
Clopidogrel
Clotrimazole
Codeine
Colchicine
Colecalciferol (with/without) calcium
Desloratadine
Desloratadine
Desloratadine
Desogestrel (oral)
Dexamethason
Dexamethason
Diazepam
Diclofenac
Diclofenac
Diclofenac
Diclofenac
Digoxin
Diltiazem
Diltiazem
Dipyridamole
Domperidone
Domperidone
Doxycycline
Doxycycline
Doxycycline
Doxycycline
Emollients (e.g. Cremor vaselini cetomacrogolis)
Emollients (e.g. Cremor vaselini cetomacrogolis)
Emollients (e.g. Cremor vaselini cetomacrogolis)
Enalapril
Epinephrine (adrenaline)
Epinephrine (adrenaline)
Esomeprazole
Ethinylestradiol/levonorgestrel (oral)
Etonogestrel (s.c. Implant)
Ferrous fumarate
Flucloxacillin
Flucloxacillin
Flucloxacillin
Fluconazole
Fluticasone
Fluticasone
Folic acid (vitamin b9)
Formoterol
Fosfomycin
Fusidic acid
Gliclazide
Glucagon
Glucose solution
Glyceryl trinitrate (nitroglycerine)
Haloperidol
Hydrochlorothiazide
Hydrocortisone
Hydrocortisone
Hydrocortisone/acetic acid

For peer review only

Hydroxocobalamin (vitamin b12)
Ibuprofen
Ibuprofen
Ibuprofen
Ibuprofen
Influenza vaccine
Insulin aspart
Insulin glargine
Ipratropium bromide
Ipratropium bromide
Isosorbide dinitrate
Isosorbide mononitrate
Ispaghula (psylla seeds)
Lactulose
Levocabastine
Levocetirizine
Levocetirizine
Levocetirizine
Levonorgestrel (iud)
Levothyroxine
Lidocaine creme
Lisinopril
Loperamide
Loperamide
Lorazepam
Losartan
Macrogol
Macrogol
Mebeverine
Medroxyprogesterone (s.c. Depot)
Menthol in aqueous cream
Menthol in aqueous cream
Metformin
Metocloperamide
Metocloperamide
Metoprolol
Metoprolol
Metronidazole
Miconazol
Midazolam (i.m.)
Mometasone
Nadroparin
Nadroparin
Naproxen
Naproxen
Naproxen
Naproxen
Nitrofurantoin
Omeprazole
Omeprazole, pantoprazole
Omeprazole, pantoprazole, esomeprazole
Ors (oral rehydration solution)
Ors (oral rehydration solution)
Oxazepam
Oxycodone
Pantoprazole
Paracetamol
Paracetamol
Paroxetine
Perindopril
Pheneticillin
Pheneticillin
Pheneticillin
Phenprocoumon
Phenprocoumon
Prednisolone
Prednisolone
Prednisolone
Prensisolone
Propranolol
Ranitidine
Risedronic acid
Rosuvastatin
Salbutamol
Salbutamol
Salbutamol
Salbutamol
Salbutamol
Salbutamol
Salmeterol
Simvastatin
Sodium chloride solution
Spironolactone
Temazepam
Tetanus toxoid
Thiamine (vit b1)
Tiotropium bromide
Tramadol
Triamcinolone
Triamcinolone/acetic acid
Trimethoprim
Trimethoprim/polymyxine b
Trimethoprim/sulfamethoxazole
Trimethoprim/sulfamethoxazole
Trimethoprim/sulfamethoxazole
Verapamil
Verapamil
Xylometazoline nasalspray
Xylometazoline nasalspray
Zolpidem

For peer review only

List of medicines	Route of administration	Route of administration	Route of administration	Route of administration
Miconazole	Oral			
Aluminium hydroxide/magnesium hydroxide	Oral			
Magnesium hydroxide	Oral			
Ranitidine	Oral			
Misoprostol	Oral			
Omeprazole	Oral			
Pantoprazole	Oral			
Esomeprazole	Oral	IV		
Mebeverine	Oral			
Atropine	IV			
Butylscopolamine	Oral			
Metoclopramide	Oral	IV	Rectal	
Domperidone	Oral			
Ondansetron	Oral	IV	Rectal	
Bisacodyl	Oral	Rectal		
Ispaghula husk	Oral			
Lactulose	Oral			
Macrogol	Oral			
Nystatin	Oral			
Oral rehydration solution (ORS)	Oral			
Loperamide	Oral			
Insulin	SC			
Insulin aspart	SC			
Protamine (novomix)	SC			
Insulin glargine	SC			
Metformin	Oral			
Gliclazide	Oral			
Glimepiride	Oral			
Acarbose	Oral			
Pioglitazone	Oral			
Sitagliptin	Oral			
Linagliptin	Oral			
Exenatide	SC			
Liraglutide	SC			
Canagliflozin	Oral			
Empagliflozin	Oral			
Repaglinide	Oral			
Cholecalciferol	Oral			
Thiamine (vit B1)	Oral	IV		
Calcium/vitamin D	Oral			
Potassium chloride	Oral			
Fenprocoumon	SC			
Acenocoumarol	SC			
Heparin	IV			

For peer review only

Enoxaparin	SC	
Nadroparin	SC	
Clopidogrel	Oral	
Dipyridamole	Oral	
Carbasalate calcium	Oral	
Prasugrel	Oral	
Dabigatran	Oral	
Rivaroxaban	Oral	
Apixaban	Oral	
Fondaparinux	SC	IV
Tranexamic acid	Oral	IV
Vitamin K	Oral	IV
Prothrombin complex concentrate	IV	
Ferrous fumarate	Oral	
Hydroxocobalamin (vitamin B12)	SC	IM
Folic acid (vitamin B9)	Oral	
Glucose solution	IV	
Potassium chloride	IV	
Digoxin	Oral	IV
Flecainide	Oral	
Amiodarone	Oral	IV
Epinephrine (adrenaline)	IM	
Nitroglycerine	SL	
Isosorbide dinitrate	Oral	SL
Isosorbide mononitrate	Oral	
Methyldopa	Oral	
Hydrochlorothiazide	Oral	
Chlorthalidone	Oral	
Furosemide	Oral	IV
Bumetanide	Oral	IV
Spironolactone	Oral	
Propranolol	Oral	
Sotalol	Oral	
Metoprolol	Oral	IV
Atenolol	Oral	
Bisoprolol	Oral	
Labetolol	Oral	IV
Amlodipine	Oral	
Nifedipine	Oral	
Barnidipine	Oral	
Lercanidipine	Oral	
Nicardipine	IV	
Verapamil	Oral	IV
Diltiazem	Oral	
Enalapril	Oral	
Lisinopril	Oral	

For peer review only

Perindopril	Oral	
Ramipril	Oral	
Fosinopril	Oral	
Losartan	Oral	
Valsartan	Oral	
Irbesartan	Oral	
Candesartan	Oral	
Simvastatin	Oral	
Pravastatin	Oral	
Atorvastatin	Oral	
Rosuvastatin	Oral	
Gemfibrozil	Oral	
Colestyramine	Oral	
Ezetimibe	Oral	
Evolocumab	SC	
Miconazole	Cutaneous	
Emollients (e.g. Cremor vaseline cetomacrogolis)	Cutaneous	
Zinc oxide	Cutaneous	
Petroleum jelly	Cutaneous	
Lidocaine creme	Cutaneous	
Menthol in hydrophilic ointment	Cutaneous	
Fusidic acid	Cutaneous	
Hydrocortisone	Cutaneous	
Triamcinolone	Cutaneous	
Betamethasone	Cutaneous	
Mometasone	Cutaneous	
Clobetasole	Cutaneous	
Clotrimazole	Cutaneous	
IUD with progestogen	IUD	
Ethinylestradiol/levonorgestrel	Oral	
Medroxyprogesterone (s.c. Depot)	SC	
Etonogestrel (s.c. Implant)	SC	
Desogestrel	Oral	
Levonorgestrel	Oral	
Estradiol	Oral	
Oxybutynin	Oral	
Solifenacin	Oral	
Tamsulosin	Oral	
Finasteride	Oral	
Betamethasone	Oral	IV
Dexamethasone	Oral	IV
Prednisolone	Oral	IV
Hydrocortisone	Oral	IV
Levothyroxine	Oral	
Glucagon	IM	

For peer review only

Doxycycline	Oral			
Amoxicillin	Oral	IV		
Feneticillin	Oral	IV		
Flucloxacillin	Oral	IV		
Amoxicillin/clavulanic acid	Oral	IV		
Meropenem	IV			
Trimethoprim	Oral			
Co-trimoxazole	Oral	IV		
Trimethoprim/sulfametoxazole	Oral	IV		
Erythromycin	Oral	IV		
Clarithromycin	Oral	IV		
Azithromycin	Oral	IV		
Gentamycin	IV			
Ciprofloxacin	Oral	IV		
Vancomycin	IV			
Fusidic acid	Oral			
Nitrofurantoin	Oral			
Fosfomycin	Oral			
Fluconazole	Oral	IV		
Rifampicin	Oral	IV		
Acyclovir	Oral	IV		
Valaciclovir	Oral			
Oseltamivir	Oral			
Tetanus toxoid	IM			
Methotrexate	Oral	IV	IM	SC
Diclofenac	Oral			
Ibuprofen	Oral			
Naproxen	Oral			
Ibuprofen	Cutaneous			
Diclofenac	Cutaneous			
Allopurinol	Oral			
Colchicine	Oral			
Alendronic acid	Oral			
Risedronic acid	Oral			
Denosumab	SC			
Fentanyl	IV			
Lidocaine	SC			
Epinephrine (adrenaline)	SC			
Morphine	Oral	IV	SC	
Oxycodone	Oral	SC		
Fentanyl	Oral	Cutaneous	Nasal	SL
Tramadol	Oral	Rectal		
Piritramide	SC			
Acetylsalicylic acid	Oral	IV		
Calcium carbasalate	Oral	IV		
Paracetamol	Oral	IV	Rectal	

For peer review only

Sumatriptan	Oral	Nasal	SC
Valproic acid	Oral	IV	
Levetiracetam	Oral	IV	
Pregabalin	Oral	IV	
Haloperidol	Oral	IV	
Clozapine	Oral		
Olanzapine	Oral		
Quetiapine	Oral		
Lithium	Oral		
Risperidone	Oral	IM	
Diazepam	Oral	Rectal	
Oxazepam	Oral		
Lorazepam	Oral	IM	IV
Temazepam	Oral		
Midazolam	Oral	IV	
Zolpidem	Oral		
Clomipramine	Oral		
Amitriptyline	Oral		
Nortriptyline	Oral		
Fluoxetine	Oral		
Citalopram	Oral		
Paroxetine	Oral		
Sertraline	Oral		
Escitalopram	Oral		
Mirtazapine	Oral		
Venlafaxine	Oral		
Metronidazole	Oral	IV	
Xylometazoline	Nasal		
Levocabastine	Ocular		
Beclomethasone	Nasal		
Budesonide	Nasal		
Mometasone	Nasal		
Fluticasone	Nasal		
Salbutamol	Inhalation		
Salmeterol	Inhalation		
Formoterol	Inhalation		
Beclometasone	Inhalation		
Budesonide	Inhalation		
Fluticasone	Inhalation		
Ciclesonide	Inhalation		
Ipratropium bromide	Inhalation		
Tiotropium bromide	Inhalation		
Tiotropium	Inhalation		
Codeine	Oral		
Clemastine	Oral	IV	
Meclozine	Oral		

For peer review only

Levocetirizine	Oral	
Desloratadine	Oral	
Chloramphenicol	Ocular	
Fusidic acid	Ocular	
Erythromycin	Ocular	
Trimethoprim/polymyxin B	Ocular	
Dexamethasone (Ocular)	Ocular	
Prednisolone (Ocular)	Ocular	
Levocabastine	Ocular	
Aluminium acetate	Ear	
Miconazole	Oral	
Hydrocortisone/acetic acid	Ear	
Triamcinolone/acetic acid	Ear	
Protamine	IV	
Naloxone	IV	
Flumazenil	IV	
Ticagrelor	Oral	IV
Cefuroxime	IV	
Cefotaxime	IV	
Ceftriaxone	IV	
Ceftazidime	IV	
Clindamycin	Oral	IV
Sodium chloride	IV	
Ringer’s lactate	IV	

For peer review only
